# Clinical Outcomes of Adjuvant Hormone Therapy in a Cohort of Patients With Infiltrating Non-metastatic Breast Cancer in a Latin American Cancer Center

**DOI:** 10.7759/cureus.27212

**Published:** 2022-07-24

**Authors:** Maria A Quintero-Ortiz, Luis Guzmán-Abisaab, Karena Garcia-Tirado, Ricardo Sanchez-Pedraza, Ivan Marino-Lozano, Carlos Lehmann-Mosquera, Javier Ángel-Aristizábal, Mauricio Garcia-Mora, Sandra E Diaz-Casas

**Affiliations:** 1 Breast and Soft Tissue Surgery, Instituto Nacional de Cancerología, Bogotá, COL; 2 Epidemiology and Public Health, Instituto Nacional de Cancerología, Bogotá, COL

**Keywords:** early breast cancer, oncologic treatment, clinical outcomes, adjuvant hormone therapy, breast cancer

## Abstract

Introduction: Breast cancer (BC) is the most commonly diagnosed cancer in women.* *This study evaluated the clinical outcomes and prognostic factors associated with disease-free survival (DFS) and overall survival (OS) in a cohort of patients diagnosed with hormone receptor-positive non-metastatic BC managed with adjuvant hormone therapy.

Methods: An observational, analytical, historical cohort study was conducted. DFS and OS rates were estimated, Kaplan-Meier survival functions were calculated, and Cox models were developed to assess the association between time to event (all-cause mortality or relapse) and hormone therapy exposure with a set of established variables.

Results: Inclusion criteria were met by 685 patients; the mean age at diagnosis was 58 years (SD=11.9 years). The most commonly used drug was tamoxifen for five years in 241 (35.7%) patients; 470 (69.6%) patients received initial therapy, 112 (16.5%) underwent switch therapy, and 93 (13.8%) had extended therapy. The factors associated with better rates of DFS and OS were early clinical stage (p=0.00), luminal A and luminal B Her2-positive biological subtypes (p=0.00), and adherence to adjuvant hormone therapy (p=0.001). Mortality rate was 0.77 deaths per 100 patients/year (95% CI, 0.51-1.2).

Conclusion: This cohort demonstrated that adjuvant hormone therapy improves DFS and OS rates in locally advanced tumors. The main factor for reducing disease progression in this cohort was adequate adherence to treatment.

## Introduction

Breast cancer (BC) is the most commonly diagnosed cancer among women. According to GLOBOCAN 2020 estimates, the incidence for BC was 47.8 worldwide, with a mortality rate of 13.6 per 100,000 persons; similar data were reported for Colombia with an incidence rate of 48.3 and a mortality rate of 13.1 per 100,000 [1-3].

The main therapeutic objective of non-metastatic BC is to cure the disease through loco-regional and systemic treatment [2]. Standard endocrine therapy consists of oral antiestrogenic medication taken daily for at least five years, which may occur with tamoxifen or aromatase inhibitors (anastrozole, exemestane, and letrozole) [1,3-5]. The duration of treatment and the agent depend on the individual clinical characteristics of each patient, such as age, menopausal status, clinical disease stage, biological subtype, and nodal involvement [3].

Scenarios for the administration of adjuvant hormone therapy are initial therapy, in which a drug is administered for five years (this scenario showed a 47% decrease in the recurrence of BC and 30% decrease in mortality); switch therapy, consisting of the prescription of a drug for the first two to three years (usually tamoxifen), continuing then with another antiestrogenic drug (aromatase inhibitors) for another two to three years to complete five years of treatment (this medication scheme showed a 20% impact on disease-free survival [DFS], while the effect was seen on overall survival [OS] only in patients with positive lymph nodes); and finally, extended therapy, in which an antiestrogenic drug is prescribed for another two to five years after completing five years of initial treatment, for a total of 7-10 years of adjuvant hormone therapy [6-10].

Hormone therapy might have side effects such as heat waves, sexual dysfunction, decreased bone mineral density, cardiovascular disease, endometrial pathology, thromboembolic disease, musculoskeletal symptoms, cognitive decline, and depression, among others, factors that can influence medication adherence [11-13]. Multiple studies have documented dropout rates of 31%-73% from adjuvant endocrine therapy in the first five years and up to 20% during the first year [14]. This study aimed to evaluate the clinical outcomes of adjuvant hormone therapy and its relationship with prognostic factors in a cohort of patients diagnosed with infiltrating non-metastatic breast cancer (INMBC) in a Latin American cancer center.

## Materials and methods

Study design

**Figure 1:**
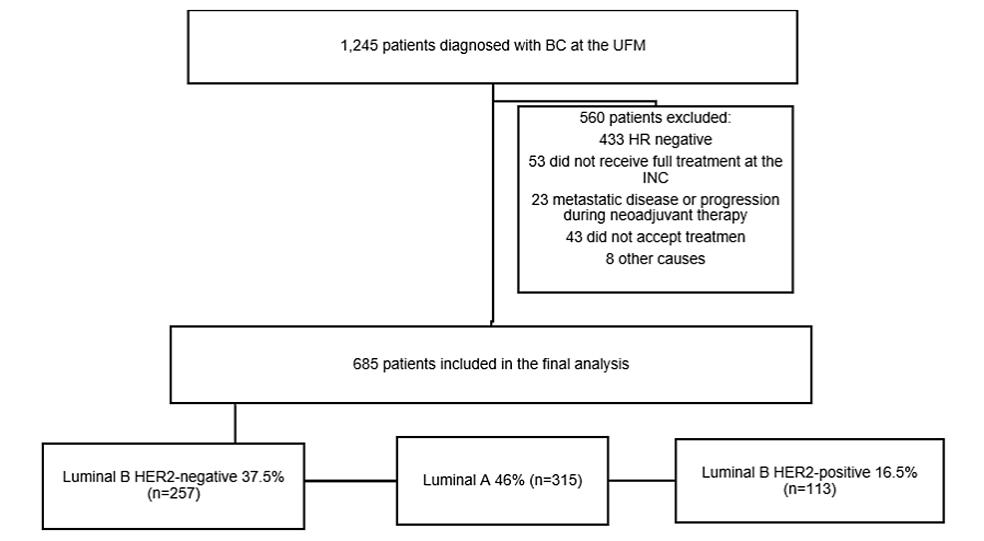
Selection of cohort patients HR, hormone receptor; UFM, Unidad Funcional de Mama; INC, Instituto Nacional de Cancerología

An observational, analytical, historical cohort study was conducted, and approved by the Ethics Committee of the Instituto Nacional de Cancerología (INC), Bogotá, Colombia. The study included 685 patients with a confirmed diagnosis of hormone receptor-positive INMBC, who were admitted to the Functional Breast Unit (Unidad Funcional de Mama, or UFM) of the INC between September 1, 2013, and August 31, 2017 (Figure 1). Patients who were hormone receptor negative, patients with metastatic breast cancer or who progressed during therapy, and patients who did not accept treatment or were treated out of the INC were excluded.

Data collection

Information on sociodemographic and clinicopathological characteristics was gathered from the UFM database and electronic medical record system (SAP, Walldorf, Baden-Württemberg, Germany). Data were collected by two of the authors and then included independently on an electronic platform designed for the storage of clinical study information (REDCap; Vanderbilt University, Nashville, TN). Subsequently, the two entries were compared, and inconsistencies were corrected by verifying values in the source. The quality and accuracy of information was evaluated by a supervisor from the INC’s Research Division.

Statistical analysis

The variables analyzed were age, menopausal status, clinicopathological and disease characteristics, medical or surgical treatment received and response to it, adverse events with adjuvant hormone therapy, causes of treatment abandonment, as well as recurrence and mortality due to BC. A descriptive analysis of categorical and nominal variables was performed using absolute and relative frequency measures; standard deviations and means were used for continuous variables. The study incorporated an analytical component that considered DFS and OS as outcomes. DFS was defined as the time between the confirmed diagnosis of BC and the date of first disease recurrence (local, regional, or distant); OS was defined as the time from the confirmed diagnosis of BC to death from any cause. The frequency of these outcomes was estimated by incidence rates measured in terms of events per 100 patient years. Rates were reported with 95% confidence intervals (CIs). Kaplan-Meier survival functions were estimated for these two outcomes. Cox proportional hazard models were used to analyze the association between outcomes and a group of variables considered risk factors. Cases of loss to follow-up or study termination without the presence of an outcome were handled as right-censoring. The proportional hazards assumption was verified by Schoenfeld residuals to test a dependent hypothesis equal to zero. The hazard ratio (HR) metric was used to interpret the Cox model coefficients. Significance values of 5% were used in all hypothesis tests. The analyses were performed using the Stata 16® statistical program (StataCorp LLC, College Station, TX).

## Results

Between September 1, 2013, and August 31, 2017, a total of 1245 patients diagnosed with breast cancer were treated for the first time at UFM. Of these patients, 685 (55%) met the inclusion criteria. The mean age of the cohort was 58 years (SD=11.9 years); 87.4% (n=599) of the tumors were ductal, with histological grade II in 67% (n=459) of the cases. The predominant biological type was luminal A in 46% (n=315), and most patients were in stage IIA (27.9%, n=191), followed by IIIB (23.4%, n=160) (Table 1).

**Table 1:** Clinicopathological characteristics of patients in the cohort

Characteristics	Number	Percentage
Age		
<35 years	10	1.4%
35-50 years	186	27.1%
>50 years	489	71.5%
T (tumor size)		
T1	132	19.2%
T2	313	45.7%
T3	63	9.2%
T4	177	25.9%
N (lymph nodes)		
N0	337	49.2%
N1	216	31.5%
N2	114	16.6%
N3	18	2.6%
Clinical stage		
I	117	17%
IA	191	27.9%
IIB	122	17.8%
IIIA	77	11.2%
IIIB	160	23.4%
IIIC	18	2.6%
Histological type		
Ductal	599	87.4%
Lobular	34	5%
Other special subtypes	52	7.6%
Biological subtype		
Luminal A	315	46%
Luminal B HER2-positive	113	16.5%
Luminal B HER2-negative	257	37.5%
Histological grade		
I	108	15.9%
II	459	67%
III	118	16.9%

The initial treatment in most patients was neoadjuvant chemotherapy (49.5%, n=339), with adriamycin, cyclophosphamide/taxanes (AC-T) being the most widely used scheme (69.9%, n=237). Initial surgical treatment was performed in 45.7% (n=313) patients. The most frequent surgical procedure was conservative surgery plus sentinel lymph node (34.6%, n=161); 236 (34.5%) patients received adjuvant chemotherapy and 599 (87.5%) underwent adjuvant radiotherapy. In relation to adjuvant hormone therapy, the most commonly used drug was tamoxifen for five years (n=241, 35.7%), followed by aromatase inhibitors in 33.3% (n=225); 470 (69.6%) patients had initial therapy, while 112 (16.5%) underwent switch therapy and 93 (13.8%) had extended therapy.

For DFS, the median follow-up was 4.3 years (95% CI, 4.1-4.6), with the minimum time being 51 days and the maximum, 7.3 years. During this follow-up time, it was possible to assess adherence to adjuvant hormonal therapy in 641 patients (44 were referred to another institution after the formulation of the drug). Eighteen (2.6%) patients discontinued adjuvant hormone therapy, the majority by own decision (66.6%, n=12) and 33.4% (n=6) for non-administration of the drug by the public health insurer. Adverse events with adjuvant hormone therapy were observed in 154 (22.5%) patients, with the main adverse event being osteoporosis evident by bone densitometry in 62.3% (n=96) of the cases. No patients in the cohort had endometrial cancer; all patients who had endometrial thickening or abnormal uterine bleeding were taken for endometrial biopsy with a report of benignity. None of the patients who had deep vein thrombosis (DVT) or pulmonary thromboembolism (PTE) died. Four (22.2%) patients discontinued treatment due to adverse events (two osteoporosis, one arthralgia, and one dyspepsia) (Table 2).

**Table 2:** Adverse events to adjuvant hormone therapy in relation to the type of administered drug

Adverse event	Tamoxifen	Aromatase inhibitor	Total
Osteoporosis evidenced by bone densitometry	23	73	96 (62.3%)
Arthralgia	4	30	34 (22%)
Deep vein thrombosis	4	1	5 (3.2%)
Abnormal uterine bleeding	5	-	5 (3.2%)
Other (rash, diarrhea, dyspepsia, transaminitis)	2	1	3 (1.9%)
Endometrial thickening	3	-	3 (1.9%)
Cataracts	2	1	3 (1.9%)
Dyslipidemia	-	2	2 (1.3%)
Pulmonary thromboembolism	2	-	2 (1.3%)
Vasomotor symptoms	-	1	1 (0.6%)

The relapse or disease progression rate was 2.9 relapse events or progression per 100 patients/year (95% CI, 2.3-3.6). During the follow-up time, there were 83 events (12%) of relapse or disease progression. Of these, 40 (48.2%) patients were treated with an aromatase inhibitor as initial therapy, 29 (34.9%) with tamoxifen, 13 (15.7%) with a switching scheme (tamoxifen + aromatase inhibitor), and 1 patient (1.2%) with gonadotropin-releasing hormone (GnRH) analog plus aromatase inhibitor. Distant involvement (67.4%, n=56) was predominant, with bone being the most compromised organ (73.6%, n=61); 21.6% (n=18) of the patients had more than two relapse sites, both local or regional, and distant. Disease progression was more frequent in luminal B HER2-negative tumors (55.4%, n=46) and in clinical stage III (68.7%, n=57). In relation to the management of patients with tumor recurrence, palliative radiotherapy to bone was used in 33.6% (n=28) of the cases, hormone therapy in 24.2% (n=20), surgery for locoregional control in 26.7% (n=22), chemotherapy in 31% (n=26), and biological or surgical therapy in 16.8% (n=14).

**Figure 2:**
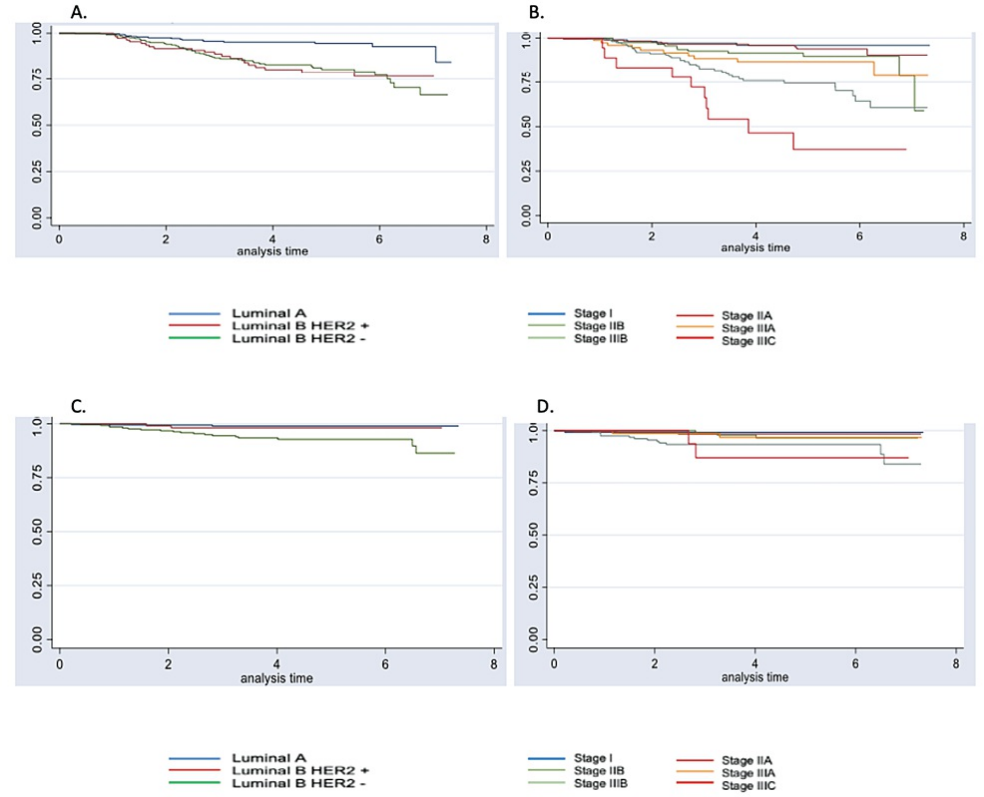
Kaplan-Meier graphs for disease-free survival by biological subtype (A) and clinical stage (B), and overall survival by biological subtype (C) and clinical stage (D)

Four variables were associated with an increased risk of relapse or disease progression: a biological subtype of BC, tumor size, nodal involvement, and clinical stage. Taking as reference patients with luminal A, T1N0, and stage I tumors, the probability of progression was greater in luminal B tumors and more advanced clinical stages (Table 3, Figure 2).

**Table 3:** Cox proportional hazards model for disease-free survival and overall survival in patients of the cohort HR, hazard ratio

Variable	Disease-free survival		Overall survival	
	HR (95% CI)	p	HR (95% CI)	p
Tumor size				
T1	1		1	
T2	2.93 (1.02-8.4)	0.044	2.5 (0.30-21)	0.36
T3	3.38 (0.95-12)	0.059	4.38 (0.39-48.4)	0.22
T4	10.3 (3.7-28.6)	0.000	10.8 (1.4-82.7)	0.02
Nodal involvement				
N0	1		1	
N1	2.76 (1.49-5.13)	0.001	4.30 (1.36-13.5)	0.012
N2	5.82 (3.15-10.7)	0.000	3.69 (0.99-13.77)	0.051
N3	13.8 (6.28-30.61)	0.000	8.6 (1.56-47.3)	0.013
Clinical stage				
I	1		1	
IIA	1.50 (0.47-4.80)	0.48	1.83 (0.19-17.64)	0.59
IIB	2.93 (0.94-9.11)	0.062	2.85 (0.29-27.48)	0.36
IIIA	4.0 (0.28-34.80)	0.019	3.15 (0.28-34.80)	0.34
IIIB	7.9 (2.81-2.19)	0.000	9.13 (1.18-70.3)	0.034
IIIC	19.5 (6.11-62.3)	0.000	12.1 (1.09-134)	0.042
Biological subtype				
Luminal A	1		1	
Luminal B HER2-negative	3.64 (1.9-7)	0.000	1.74 (0.29-10.44)	0.542
Luminal B HER2-positive	3.64 (2.06-6.44)	0.000	7.26 (2.13-24)	0.001
Abandonment of adjuvant hormone therapy				
Yes	1		1	
No	0.44 (0.19-1.03)	0.059	0.12 (0.08-0.43)	0.001

For the analysis of overall survival, the 685 patients included in the study contributed a total of 2993.2 years of follow-up. The median follow-up was 4.6 years (95% CI, 4.3-4.7), with the minimum time being 51 days and the maximum being 7.3 years. During this follow-up time, there were 23 (3.36%) deaths. The mortality rate was 0.77 deaths per 100 patients/year (95% CI, 0.51-1.2). Again, biological subtypes different from luminal A, nodal involvement, and advanced clinical stages were associated with an increased risk of death (Table 3, Figure 2). On the other hand, good adherence to adjuvant hormone therapy was shown as a protective factor for OS (p=0.001) (Table 3).

## Discussion

This cohort is similar to other studies that have analyzed the clinicopathological characteristics of patients with INMBC; 71% of the patients had positive hormonal receptor. The mean age at diagnosis was slightly lower (58 years) than the mean age at diagnosis of BC in the general population, reported between 62 and 64 years. Most of the patients in this cohort had locally advanced tumors (stages IIB, IIIA, IIIB, and IIIC), which constitute 53.9%; T4 tumors were present in 25.9%, which is very different from the results reported in previous studies where T4 tumors were present in 2.7%-8% [9,15,16]. Likewise, the percentages of N2 (16.6%) and N3 (2.6%) tumors were reported to be only 3.3%-15% in other studies [9,16,17]. It should be noted that the percentage of disease relapse in this group (12%) was lower than that those reported in other series (14.2%-79%) [16,18-20]. This advanced presentation of cases is because patients referred to the INC have limited access to health services and screening tests.

The factors associated with DFS and OS were the same as those reported in previously conducted clinical trials, i.e., tumor size, nodal involvement, clinical stage, and biological subtype [9,15,17]. Good adherence to hormonal treatment was associated with better oncological outcomes in this cohort [9,15,17].

The main secondary event in this study was osteoporosis in 62.3% of the patients, showing a higher incidence than in other studies where it ranged from 1.3% to 21% [9,17,18,21]. Arthralgia occurred in 22% of the cases, which is lower than that presented in the Duration of Adjuvant Aromatase (DATA) study, which was 49% [9]. It is very similar to the 27.8% reported in the Arimidex, Tamoxifen, Alone or in Combination (ATAC) study, and much higher than what has been reported in other studies (5.4-14%) [16-18,21].

Abnormal uterine bleeding and endometrial thickening were uncommon adverse events (3.2% and 1.9%, respectively) when compared to data reported in the literature (5.5%-8.2%) [16,21]. In this cohort, no cases of endometrial cancer were found, which is different from what has been reported in other studies (0.5%-11.3%) [16,18]. Other important outcomes that occurred in this population were PTE, DVT, vasomotor symptoms, and cataracts, which coincide with the reported data [11].

This cohort showed a low percentage of abandonment of adjuvant hormone therapy (2.6%), unlike that reported by other authors (11.7% and 47.1%) [9,11,17,20]. This difference can be explained by the adequate and strict follow-up of patients by the breast and clinical oncology services of the INC. The percentage of patients who abandoned treatment due to the presence of secondary events was low (22.2%), with the patient’s decision being the most frequent cause of abandonment (66.6%), followed by the non-administration of the drug by the public health insurer (33.4%). This is due to the organization of the health system in Colombia, given greater difficulties to obtain continuous access to the authorization of medicines by insurers in the subsidized regime.

## Conclusions

Hormone therapy prolonged disease-free survival and overall survival among patients with non-metastatic hormone receptor-positive breast cancer even in the locally advanced stage subgroup. The main factor for reducing disease progression in this cohort was adequate adherence to treatment.
